# Impact of the Static Magnetic Field on Growth, Pigments, Osmolytes, Nitric Oxide, Hydrogen Sulfide, Phenylalanine Ammonia-Lyase Activity, Antioxidant Defense System, and Yield in Lettuce

**DOI:** 10.3390/biology9070172

**Published:** 2020-07-17

**Authors:** Arafat Abdel Hamed Abdel Latef, Mona F. A. Dawood, Halimeh Hassanpour, Maryam Rezayian, Nabil A. Younes

**Affiliations:** 1 Biology Department, Turabah University College, Turabah Branch, Taif University, Taif 21995, Saudi Arabia; 2 Botany and Microbiology Department, Faculty of Science, South Valley University, Qena 83523, Egypt; 3 Botany and Microbiology Department, Faculty of Science, Assiut University, Assiut 71516, Egypt; mo_fa87@aun.edu.eg; 4 Aerospace Research Institute, Ministry of Science Research and Technology, Tehran 14665-834, Iran; hassanpour@ari.ac.ir; 5 Department of Plant Biology, and Center of Excellence in Phylogeny of Living Organisms in Iran, School of Biology, College of Science, University of Tehran, Tehran 14155-6455, Iran; maryamrezayian@ut.ac.ir; 6 Horticulture Department, Faculty of Agriculture, Al-Azhar University, Assiut Branch, Assiut 71524, Egypt; nabil.ali@azhar.edu.eg

**Keywords:** antioxidant system, crop yield, lettuce, osmolytes, oxidative damage, static magnetic field

## Abstract

Magnetic fields are an unavoidable physical factor affecting living organisms. Lettuce seeds (*Lactuca sativa* var. cabitat L.) were subjected to various intensities of the static magnetic field (SMF) viz., MF0 (control), SMF1 (0.44 Tesla (T), SMF2 (0.77 T), and SMF3 (1 T) for three exposure times (1, 2, and 3 h). SMF-treated seedlings showed induction in growth parameters and metabolism comparing to control. All photosynthetic pigments were induced markedly under SMF, especially chlorophyll a. SMF at different intensities boosted osmolytes, non-enzymatic antioxidants, and the phenylalanine ammonia-lyase activity over non-magnetized seedlings. Oxidative damage criteria viz., hydrogen peroxide, superoxide radical, and lipid peroxidation, as well as polyphenol oxidase activity, were kept at low values under SMF-treated seeds relative to control, especially SMF2. Electron donors to antioxidant enzymes including nitrate reductase, nitric oxide, and hydrogen sulfide induced via SMF exposure and consequently the activities of superoxide dismutase, glutathione-S-transferases, catalase, and peroxidases family enzymes were also stimulated under SMF, whatever the intensity or the exposure period applied. All these regulations reflected on the enhancement of lettuce yield production which reached 50% over the control at SMF3. Our findings offered that SMF-seed priming is an innovative and low-cost strategy that can improve the growth, bioactive constituents, and yield of lettuce.

## 1. Introduction

Lettuce (*Lactuca sativa* var. *cabitat L*.) is a leafy green vegetable that belongs to Asteraceae family. It is cultivated extensively for food consumption and as a source of folate, vitamins, and minerals. Lettuce is a suitable candidate for the improvement of agricultural traits, containing the transfer of nutrients and bioactive compounds valuable to human health. So, providing a good managing strategy for lettuce-growing culture including microbial resistance [1], environmental stress tolerance [2,3], yield enhancement [4], and increased active biomolecules [5] is crucially important in the agriculture sector. Today, plant productivity is an ever-growing issue that attracts researchers worldwide to apply eco-friendly innovation techniques for crop development. Various application techniques have emerged to promote plant development, including using synthetic plant protectants, biofortification of soils with microbes, manure application, and nanotechnology. Use of a magnetic field (MF) is an incoming approach to improve plant growth and productivity.

MFs are an unavoidable physical factor affecting living organisms due to the industrial revolution and many human-made practices. Using physical stresses including MFs has been recently considered for the induction of plant growth, yield, and accumulation of secondary metabolites, as stresses have a minor perilous effect for the environment [6]. Living organisms, including plants, produce and utilize various electrical domains during their functioning, as trans-membrane, electric, action, or streaming potential; thus, an MF has an influential impact on the progress and metabolism of plants [7]. Physically, magnetism affects the biological system via various proposed mechanisms, including the classical and quantum-oscillator mechanism, the cyclotron-resonance system, the joint reaction of bound ions quantum yield and electrons, excitations of coherent-quantum, and torsion fields–induced bioeffects associated with MFs. These effects also include metastable states of liquid water induced bio-active effects, free-radical pathways and other “spin-mechanisms,” the “parametric-resonance” mechanism, “stochastic resonance” as an amplifier model in magnetobiology and other random pathways, phase transitions in biophysical models affecting liquid crystal ordering, bifurcation behavior of solutions of non-linear chemical kinetics equations, radio-technical concepts, in which biological systems are portrayed as equivalent electric circuits, and macroscopic charged vortices in the cytoplasm, and the interplay between these mechanisms, are also present [8]. These effects are translated by plants as alterations in morphogenesis, biochemical reactions inside the cells. In this regard, MFs can influence living organisms by affecting the activity of free radicals and altering ionic transports in cell membranes. MFs can also change electrical properties and permeability of the membrane and metabolic pathways in plant cells [9].

To assess the impact of magneto-priming on plants, different kinds of MFs such as extremely low-frequency magnetic field, static magnetic field (SMF), or pulsed electromagnetic treatments were employed for varied durations and/or frequencies of exposure [10]. The SMF is characterized by low unstable parameters relative to the other types of MFs, which facilitate its application on biological systems. In this regard, SMF exerts moderate effects on living cell beings comparing to time-variable of the magnetic field and many of their alterations are profitable [11]. Pre-exposure of seeds to MFs as a physical technique in agriculture and could be an innovative discipline to enhance the course of germination and seedlings vigor, morphogenesis, and increasing crop yield without harmful impact on the ecosystem. Different cellular components and organelles including mitochondria, cell membranes, protein, and DNA change their electromagnetic behavior under SMF [12,13], hence affecting various physiological and biochemical responses in the cells. So far, many studies have been conducted to study the effect of MF on living organisms, but the exact mechanism, especially in plants, is still largely unclear. The stimulations of growth in crops under precise static magnetic conditions have been confirmed, but extensive work is still needed to highlight the mechanisms of magnetic field therapy in plants. For example, SMF priming at 1 mT for 1 h promoted germination, growth, and protein content in canola seeds as compared to control, and higher intensity diminished the mentioned parameters [14]. Photosynthesis level, chlorophyll content, and growth rate were promoted in soybean seeds exposed to 200 mT [15]. SMF at 30 mT improved the taxol accumulation in hazel (*Corylus avellana*) cell suspension [16]. Sahebjamei et al. [9] demonstrated that MF improved the antioxidant enzyme activity in tobacco cell suspension. De Souza et al. [17] reported that non-uniform MF significantly improved the root and shoot lengths and weights at germination and the vegetative stages and improved the bulb yield in the bulb formation and maturity stage of onion. Youssef and Kamer [18] reported the increase of growth parameters and nutrient content in lettuce cultivated in magnetized-hydroponic nutrient solution at 1.45 Tesla (T).

Although the bio-effects of SMF have been experienced for many plants on growth and biochemical responses on the germination and vegetative stages during the last 25 years, the literature is still very scanty, and there has been little input regarding the mechanistic effects of SMF on the cultivated plants. Few works have included the effect of SMF on the reproductive stage of cultivated plants such as lettuce. Thus, in the present study, we attempted to explain the impact of SMF on the growth and response mechanisms of lettuce plants. Therefore, we arranged a series of experiments to investigate the possible promotion effect of SMF on the growth features, the photosynthetic responses, osmolyte accumulation, non-enzymatic antioxidants, phenylalanine ammonia-lyase activity and electron donors to antioxidant defense system, as well as crop yield production.

## 2. Materials and Methods

### 2.1. Magneto-Priming Treatment and Experimental Setup

The lettuce seeds (*Lactuca sativa* var. cabitat L.) were purchased from Mecca TRADE Co., Egypt.

The magnetic field was applied using a locally designed homogenous SMF generator with a power supply (PHYWE power supply, Germany) for producing various SMF intensities up to 2 T. This system has consisted of copper wire (0.5 mm in diameter), wrapped around an electrical coil in the dimensions 3 wide × 3 long × 6 cm height. The electrical current ran through the coil, the magnetic field was adjusted by using a Rheostat (PHYWE, Germany), and the used current in the coil was detected and measured by using a conventional multimeter. Seeds were put in the coil center to get uniform intensity throughout the container, and the intensity of MF was measured by the Tesla meter (PHYWE, Germany) accompanied by a sensor inside the core of the coil.

The seeds were subjected to four intensities of SMF—MF0 (control), MF1 (0.44 T), MF2 (0.77 T), and MF3 (1 T)—and each SMF was applied at three durations (1, 2, and 3 h).

Non-treated seeds and magneto-primed seeds were sown in trays filled with Peetmoss and then were organized in a complete randomized block design in three replications. The trays were placed in a greenhouse at 25 ± 2 °C with three replicates for each treatment (60 seeds per treatment). The trays received 150 mg L^−1^ NPK solution 20: 20: 20 as a fertilizer twice a week. After six weeks, seedlings were harvested for morphological and biochemical analysis.

For crop yield production, the seedlings (six weeks) were transplanted to open field at a private farm in Al-Atwany, Edfu City, Aswan, Egypt which located at 24.0889° N, 32.8998° E. The seedlings were cultivated at 30 cm away in one side of the edges. The plot area was 11.2 m^2^, which composed of six rows (4 m length and 50 cm in width). The experiments were managed in a complete randomized block design in three replications. Normal cultural practices were performed as suggested for the conventional lettuce culturing based on the instructions of the Egyptian Ministry of Agriculture. The soil physical and chemical characteristics were pH (7.4), CaCO_3_ (1.62%), Na^+^ (6.5%), N (0.032%), P (0.0054%), NH_4_^+^ (48%) and EC (2.4 dS m^−1^). After eight weeks of transplanting, the heads were collected to estimate crop yield production.

### 2.2. Growth Parameters and Pigments Content

During the seedbed period, 10 seedlings for each treatment were considered at 40 days post-sowing to determine shoot and root length, shoot and root dry weight (DW), number of lateral roots, and stem thickness. Lettuce seedlings after harvesting were rinsed with deionized water and placed on paper towels before weighting. Samples were oven-dried at 40 °C for 72 h. Chlorophyll (Chl) a and b and carotenoids (Car) were detected in the solution of suspending fresh leaves in ethyl alcohol (95%) for 12 h, and then the absorbance readings were recorded at 663, 644, and 452 nm [19].

### 2.3. Compatible Osmolyte Contents

The soluble sugars were detected based on the method of anthrone-sulphuric acid [20,21] using glucose as a standard and the absorbance reading was followed at 620 nm. Proline was determined in the supernatant result from the maceration of fresh leaves in 5-sulfosalicylic acid [22]. Total soluble proteins in leaves were quantified using alkaline and Folins reagents, where absorbance was detected at 750 nm [23]. Total free amino acids in leaves extract were determined using ninhydrin-citric acid reagent, and the absorbance was elicited at 570 nm [24].

### 2.4. Reactive Oxygen Species (ROS) and Lipid Peroxidation Content

The hydrogen peroxide (H_2_O_2_) level of fresh leaves was quantified spectrophotometrically at 415 nm in cold acetone extract of leaves + titanium dioxide-sulfuric acid reagent [25]. Superoxide radical (O_2_^−^) content was measured following the nitrite creation from hydroxylamine at 530 nm [26]. Lipid peroxidation in fresh leaf samples was detected through the method of thiobarbituric acid via scanning malondialdehyde production [27].

### 2.5. Nitrate Reductase (NR), Nitric Oxide (NO), and Hydrogen Sulfide (H_2_S) Content

The activity of NR was quantified in the incubation medium of fresh leaves in K-phosphate buffer + KNO_3_. 1-Naphthyl-ethylenediamine dihydrochloride and sulfanilamide were added to the last incubation medium to elicit nitrite formation at 540 nm [28].

NO content was quantified by mixing the supernatant of fresh leaves homogenized in acetate buffer with Griess reagent [29,30].

H_2_S was detected in the supernatant of frozen leaves macerated in K-phosphate buffer and ethylenediaminetetraacetic acid, then mixed with 5,5′-dithiobis(2-nitrobenzoic acid), and the absorbance readings were measured at 415 nm [31].

### 2.6. Enzymatic Antioxidant Assay

For the determination of enzymatic antioxidant activities, fresh leaves were homogenized in liquid nitrogen, and total proteins were measured as defined by Ahmad et al. [32]. Enzyme activity of superoxide dismutase (SOD; EC 1.15.1.1), ascorbate peroxidase (APX; EC 1.11.1.11), catalase (CAT; EC 1.11.1.6), peroxidase (POD; EC 1.11.1.7), polyphenol oxidase (PPO; EC 1.10.3.1), glutathione peroxidase (GPX; EC 1.11.1.9), and glutathione-S-transferase (GST; EC 2.5.1.18) were assessed through the methods [33,34,35,36,37,38,39], respectively.

### 2.7. Non-Enzymatic Antioxidant Assay

#### 2.7.1. Total Phenolic and Flavonoid Content

Total phenolics were quantified in methanolic leaves extract through the addition of sodium carbonate and Folin–Ciocalteu reagent [40] using gallic acid as a standard. Total flavonoids were quantified in methanolic extract spectrophotometrically at 510 nm using aluminum chloride + sodium hydroxide + sodium nitrite [41], and quercetin was used as a standard curve.

#### 2.7.2. Anthocyanin Content

Anthocyanin content was measured using 1% HCl *v/v* acidified methanol. Fresh leaves were homogenized in the extraction solution, centrifuged at 18,000× *g* at 4 °C for 15 min, and stored in darkness for 5 h at 5 °C. The amount of anthocyanin was quantified at 550 nm [42].

#### 2.7.3. Ascorbic Acid (ASA) and Reduced Glutathione (GSH) Content

Here, 0.5 g fresh leaves were mixed in 5% trichloroacetic acid, centrifuged at 11,500× *g* at 4 °C for 15 min, and the supernatant was utilized for quantification of ascorbic acid (ASA) using Folins reagent [43] and reduced glutathione (GSH) using Ellman’s reagent [44].

#### 2.7.4. α-Tocopherol Content

Fresh leaves were grounded in 8 mL chloroform, centrifuged at 4 °C for 15 min, and the supernatant was applied for measuring α-tocopherol [45] through 2,2′-dipyridyl and ferric chloride reagents.

### 2.8. Phenylalanine Ammonia-Lyase (PAL) Activity

The previously prepared enzyme extract was incubated with borate buffer + phenylalanine and then the absorbance of the formed trans-cinnamic acid was followed at 290 nm [46].

### 2.9. Crop Yield Production

After 8 weeks from transplanting, the lettuce heads were collected to calculate crop yield in ton/hectare.

### 2.10. Statistical Analysis

The experiments were performed in a randomized complete block design. The data examined by the analysis of variance (ANOVA) with SPSS software (version 18) and Duncan’s multiple range test was conducted at the *p* ≤ 0.05 level of significance. Data displayed as mean ± standard errors (SEs) of three independent replicates of each treatment. Principal component analysis (PCA) was done using XLSTAT (2016) software.

## 3. Results

### 3.1. Impact of Different Intensities of SMF for Three Exposure Periods on Growth Criteria and Yield

The results shown in Table 1 indicated that SMF significantly increased all morphological traits in terms of shoot length, root length, shoot DW, root DW, number of lateral roots, and stem thickness as compared to non-magnetized plants. The maximum enhancement of shoot length (26.54%) and shoot DW (245.208%) were detected at SMF3 for 1 h. Root length was induced highly significantly by SMF and the promotion of SMF was more prominent at SMF1 for 3 h (74.80%). The highest incremental of root DW was recorded in response to the exposure to SMF1 for 2 h, SMF2 for 3 h, and SMF3 for 1 h. Stem thickness was significantly increased whatever the magnetic field applied and increased 2.5-fold at SMF2 for 2 h and 3 h. Interestingly, the improvement of growth criteria via magnetization of seed was not restricted to growth criteria, and the enhancement extended to crop yield production. The increment of crop yield was significant under SMF treatments and an increase of 47.32% and 56.85% compared to control was observed at SMF2 and SMF3 for 3 h over the control plants, respectively (Table 1).

### 3.2. Impact of Different Intensities of SMF for Three Exposure Periods on Pigments Content

**Figure 1:**
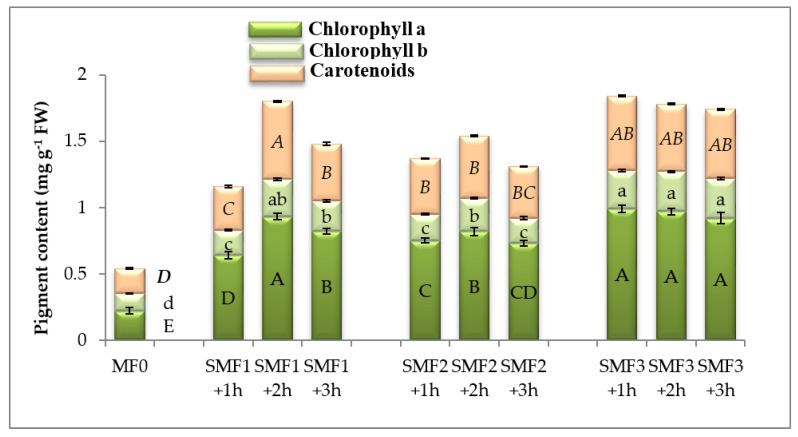
Effect of different intensities of the static magnetic field (SMF) for three exposure periods on chlorophyll a and b and carotenoids contents of lettuce. Different letters of the same format indicate statistically significant values following Duncan’s multiple range test at *p* < 0.05. Bars represent means of three (*n* = 3) replicates with standard errors (SEs). SMF0 (control), SMF1 (0.44 T), SMF2 (0.77 T), and SMF3 (1 T). FW—fresh weight; T—Tesla; h—hour.

SMF significantly induced active photosynthetic pigment contents in lettuce plants, especially Chl as compared to Chl b and carotenoids. Seed magnetization at SMF3 showed the highest positive impact on Chl a and Chl b contents (Figure 1). A marked rise in Car content was observed in lettuce leaves subjected to SMF treatment, and the maximum effect (3.12-folds) was shown at SMF1 for 2 h over the control plants (Figure 1).

### 3.3. Impact of Different Intensities of SMF for Three Exposure Periods on Osmolytes

The data represented in Table 2 illustrated that SMF induced a significant enhancement of the total soluble sugars content, and this effect was much more significant (ca. 109.16%) at MF1 and MF2 for 3 h. Total soluble proteins content significantly reinforced at different intensities of SMF, and the maximum increase was 208.62% obtained at SMF2 for 2 h. Also, the content of total free amino acids enhanced significantly at SMF 1 and 2 at different times, and the highest contents were observed at 3 h of treatment (Table 2). The osmolyte proline biosynthesis was also affected by seed magnetization, as presented in Table 2. SMF-induced proline content accumulation which was much more so at MF2 for 1 h and 2 h (*ca*. 5.89-fold) compared to non-magnetized seeds. In general, the cumulative dose of SMF3 at 3 h showed the lowest accumulations of osmolytes.

### 3.4. Impact of Different Intensities of SMF for Three Exposure Periods on ROS and Oxidative Damage Trait

**Figure 2:**
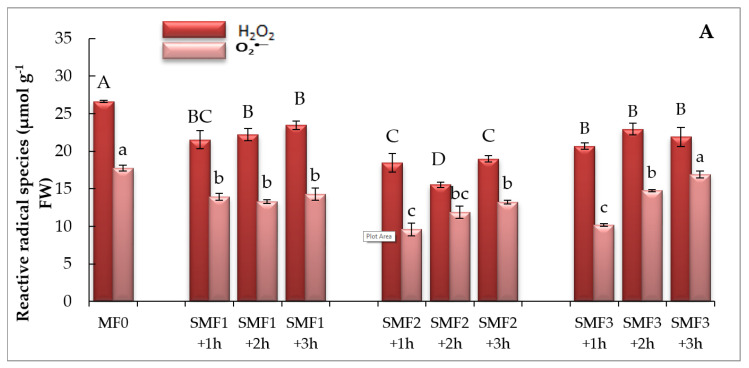

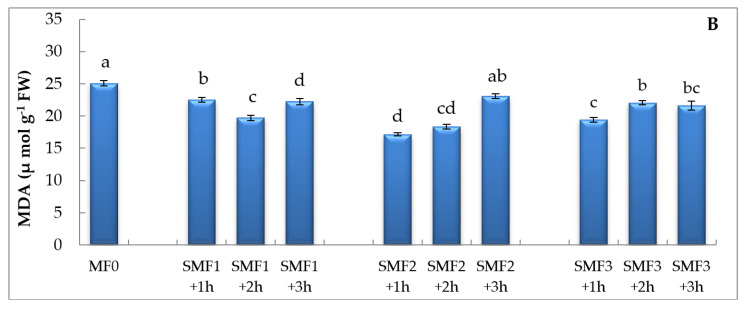
Effect of different intensities of the static magnetic field (SMF) for three exposure periods on the (**A**) H_2_O_2_ and O_2_^−^ and (**B**) malondialdehyde (MDA) contents of lettuce. Different letters indicate statistically significant values following Duncan’s multiple range test at *p* < 0.05. Bars represent means of three (*n* = 3) replicates with standard errors (SEs). SMF0 (control), SMF1 (0.44 T), SMF2 (0.77 T), and SMF3 (1 T). FW—fresh weigh; T—Tesla; h—hour.

The data displayed in Figure 2 includes the change in ROS under magnetism and control treatments. SMF caused a considerable decline in H_2_O_2_ and O_2_^−^ production as compared to control plants (Figure 2). The minimal foliar content of H_2_O_2_ and O_2_^−^ were observed at SMF2 for 1 h and 2 h, respectively (Figure 2). The data also declared that lipid peroxidation in terms of MDA content as a marker of oxidative stress was tested for magnetized and non-magnetized plants. SMF significantly reduced MDA values, relative to the control, with a maximum reduction of 31.69% under SMF2 treatment for 1 h (Figure 2).

### 3.5. Impact of Different Intensities of SMF for Three Exposure Periods on NR Activity, NO, and H_2_S Content

**Figure 3:**
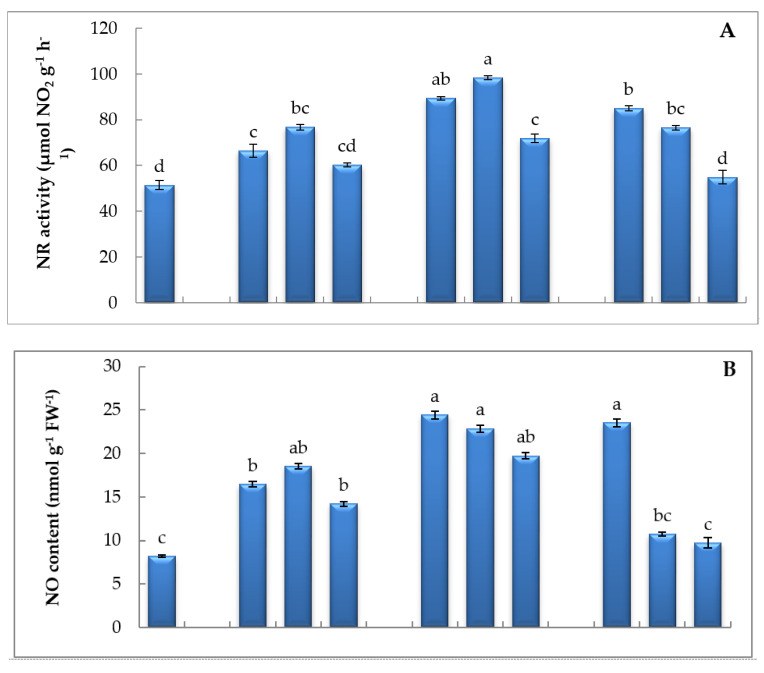

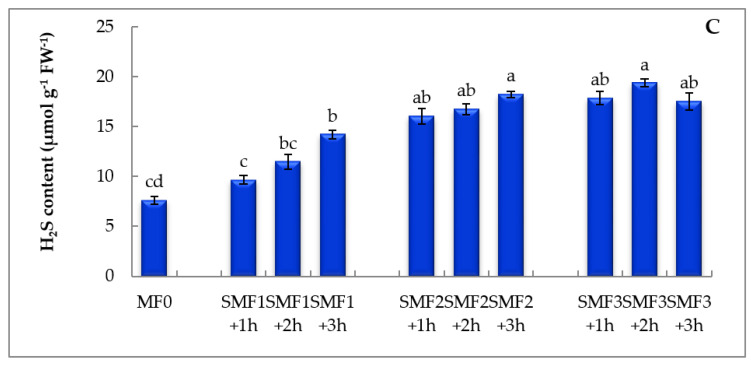
Effect of different intensities of the static magnetic field (SMF) for three exposure periods on (**A**) nitrate reductase (NR) activity, (**B**) nitric oxide (NO), and (**C**) hydrogen sulfide (H_2_S) contents of lettuce. Different letters indicate statistically significant values following Duncan’s multiple range test at *p* < 0.05. Bars represent means of three (*n* = 3) replicates with standard errors (SEs). SMF0 (control), SMF1 (0.44 T), SMF2 (0.77 T), and SMF3 (1 T). FW—fresh weigh; T—Tesla; h—hour.

The application of SMF on seeds exhibited triggering of NR activity, and the paramount increase was recorded at MF2 for 2 h by about 47.69% relative to control (Figure 3). The application of magnetic therapy activated the pool of NO production whatever the exposure dose or time, and the highest increment was manifested at MF2 for 1 h and 2 h in comparison to other intensities (Figure 3). Moreover, SMF induced H_2_S content exacerbation, especially at SMF2 and SMF3 during the different exposure periods (Figure 3).

### 3.6. Impact of Different Intensities of SMF for Three Times on Enzymatic Antioxidants

**Figure 4:**
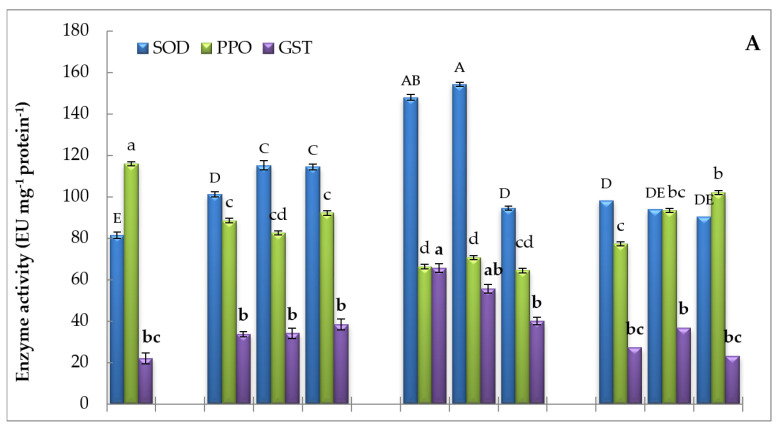

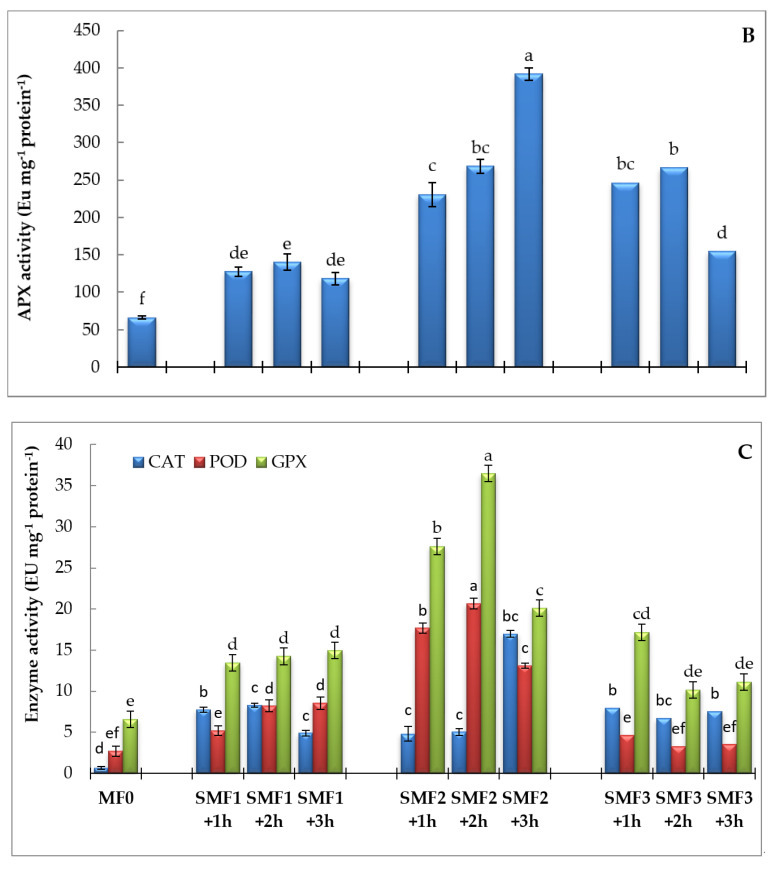
Effect of different intensities of the static magnetic field (SMF) for three exposure periods on the activities of (**A**) superoxide dismutase (SOD), polyphenol oxidase (PPO), and glutathione-S-transferase (GST); (**B**) ascorbate peroxidase (APX); and (**C**) catalase (CAT), peroxidase (POD), and glutathione peroxidase (GPX) of lettuce. Different letters of the same format indicate statistically significant values following Duncan’s multiple range test at *p* < 0.05. Bars represent means of three (*n* = 3) replicates with standard errors (SEs). SMF0 (control), SMF1 (0.44 T), SMF2 (0.77 T), and SMF3 (1 T). T—Tesla; h—hour.

The alternation of antioxidant enzyme activities showed dissimilar trends under SMF treatment as shown in Figure 4. The magnetic field treatment up-regulated superoxide and hydrogen peroxide metabolizing enzymes. As represented in Figure 4A–C, the SOD, GST, CAT, APX, GPX, and POD activities were augmented in lettuce plants under the different durations of SMF with different magnitudes. The maximal activity of SOD, POD, GST, and GPX was registered at MF2 during the exposure periods of 1 h and 2 h (Figure 4A,B), whereas the upmost activities of APX and CAT were shown at MF2 for 3 h (Figure 4B,C). PPO activity was deregulated by SMF exposure, as the magnetized seedlings exhibited a reduction of PPO activity by magnetization, and the maximum decrease presented at MF2 for 1, 2, and 3 h relative to control (Figure 4A). Overall, it seems that MF2 at different times caused more induction of antioxidant enzyme activities compared to control and other SMF intensities.

### 3.7. Impact of Different Intensities of SMF for Three Exposure Periods on Secondary Metabolism, Non-Enzymatic Antioxidants, and PAL Activity

Secondary metabolism is also affected under the interactive effect of magnetic field application on lettuce seeds. As represented in Table 3, SMF treatment caused a significant enhancement in phenolic content in comparison to control plants. The highest phenolic content was recorded at SMF1 under the three applied durations. The data declared that the cumulative dose of SMF for 3 h exhibited the highest accumulation of phenolic content. SMF also had a stimulatory role on flavonoid biosynthesis, where exacerbation of its content was monitored whatever the SMF intensity applied or duration exposed. The highest accumulation was denoted for the cumulative dose (2 h) whatever the intensity applied. Anthocyanin is another secondary metabolite that showed the bio-stimulation effect under SMF (Table 3). In this sense, the leaves of control plants recorded anthocyanin content by about 0.07 µg g^−1^ FW, whilst the highest values of anthocyanin were 0.22 and 0.25 at SMF3 (2 h) and SMF2 (3 h), respectively (3.5-fold higher relative to control).

Non-enzymatic antioxidants in terms of low molecular weight compounds as ASA, *α*-tocopherol, and GSH content were also involved in the current investigation. ASA and GSH contents were induced in the plants exposed to SMF comparing to control (Table 3). Exposure to MF2 for 2 h led to a significant increase in ASA (68.31%) and GSH (69.74%) contents as compared to control. SMF at different intensities and durations significantly induced *α*-tocopherol contents as compared to control, and an increment of 164.99% was observed as a result of seed exposure to SMF2 for 3 h (Table 3).

The phenolic-synthesizing enzyme PAL was also affected by SMF. The promotion of PAL activity was also witnessed under all intensities of SMF, and the highest activity was identified at MF2 for 2 h (129.12%) as compared to control (Table 3).

### 3.8. PCA Analysis of Different Variable Relationships in Lettuce under SMF Exposure

**Figure 5:**
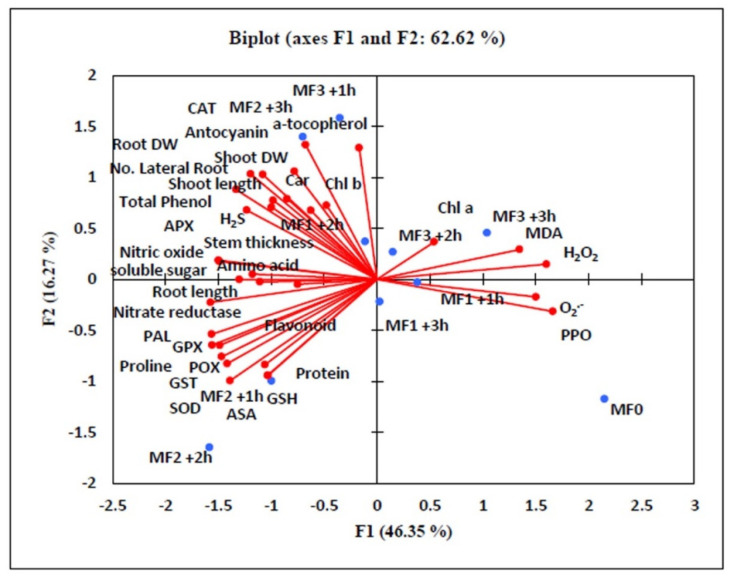
Effect of the static magnetic field (SMF) on the principal component analysis (PCA) of different variable relationships in lettuce. The PCA test identified 62.62% of the total variation, and axis 1 and axis 2 accounted for 46.35% and 16.27%, respectively. Growth parameters displayed positive correlations with H_2_S, NO, nitrate reductase, osmotic adjustments, and enzymatic and non-enzymatic antioxidants. DW (dry weight), Chl (chlorophyll), Car (carotenoids), H_2_O_2_ (hydrogen peroxide), MDA (malondialdehyde), NR (nitrate reductase), NO (Nitric oxide), hydrogen sulfide (H_2_S), SOD (superoxide dismutase), PPO (polyphenol oxidase), GST (glutathione-S-transferase), APX (ascorbate peroxidase), CAT (catalase), POD (peroxidase), GPX (glutathione peroxidase), ASA (ascorbic acid), GSH (reduced glutathione), PAL (phenylalanine ammonia-lyase), SMF0 (control), SMF1 (0.44 T), SMF2 (0.77 T), SMF3 (1 T), T (Tesla), and h (hour).

The data represented in Figure 5 showed the correlation analyses calculated based on Pearson’s coefficient. Shoot DW and root DW as growth parameters displayed a positive correlation with CAT, SOD, POD, APX, GST, GPX, NR, NO, H_2_S, carotenoid, Chl b, proline, soluble sugar, amino acid, anthocyanin, phenol, *α*-tocopherol, and PAL. On the other hand, these growth traits declared a negative correlation with ROS (H_2_O_2_, and O_2_^−^) and lipid peroxidation calculated in terms of MDA content. The figure also denotes that H_2_O_2_, O_2_^−^ and MDA displayed negative correlations with enzymatic and non-enzymatic antioxidants, proline, soluble sugar, and amino acid. On the other hand, a positive correlation was detected between secondary metabolites and PAL activity. Another positive correlation was recorded between electron donors (H_2_S, NO, and NR) and antioxidant enzymes.

## 4. Discussion

Plant motivation is a way to achieve better plant growth and is a communal practice applied in modern and environmentally friendly agriculture [47]. Biophysical techniques such as magnetic and electromagnetic applications can be outstanding and environmentally sound methods in the agriculture sector and enhance the productivity and quality of crops produced in a low-cost method. The data of the present work displayed the positive impacts of SMF on lettuce growth and crop yield. Besides, the data recommended that the bio-impact of SMF treatment could be related to the strength of the SMF and exposure period. SMF induces growth by activating the production of protein and activates the root tropism by changing the amyloplasts direction in the statocyst of root cap cells [48]. It was revealed that SMF stimulated mitotic activity in root meristems of plants [49] and increased the growth and yield [50]. Furthermore, the effect of MF on the spatial distribution of ion fluxes along with the plants’ organs, cytoplasmic streaming, and the cell growth process jointed with intracellular mass and charge transfer [51]. This could interpret the enhancement of roots morphogenesis (lengths, dry weight, and the number of lateral roots) under SMF, and the intensities 0.77 T and 1 T achieved this equilibrium. The biostimulatory impact of SMF on the germination, growth, and yield was apparent in other plants [15,52].

The magnetic field influenced plastids due to their paramagnetic character. In this regard, MF enhances plant energy in plants and spreads this energy to biomolecules, thereby catalyzing the metabolic pathways accelerating the germinability of seeds. The free radicles inside these metabolically active cells have unpaired electrons with a magnetic field that can be oriented under an external MF causing absorption of microwave energy. This energy is transformed into chemical energy utilized by cells in the activation of biochemical pathways kinetics and magnetized plants [53]. This response might be connected with the increment of photosynthesis, as proposed by a rise in Chl a, Chl b, and carotenoids content by SMF. This finding was observed because the magnetization affected the transmembrane transport of Fe via apoplectic pH regulation; part of this activated Fe participates in chlorophyll biosynthesis, while the remaining part was released into the cell matrix [54]. The exacerbation of active photosynthetic pigment by different magnetic fields positively enhances C metabolism. It can be presumed that the enhancement of chlorophyll content as the key photosynthetic pigment in cells might maybe result in additional carbohydrate construction. The soluble sugars play a pivotal role in the osmotic regulation and conserves the proteins and cell membranes against dehydration [55]. Soluble sugars may be contributed to control the defense against different ROS-producing stresses [56]; thus, the magnetized lettuce plants had better metabolic pools, which allow their best growing under changeable conditions of the open field relative to non-magnetized ones. As germinating plants are sessile, there might be a distinguishing effect of the terrestrial magnetism on the growth and physiology of plants [57]. Thus, magnetoreception by lettuce seeds might induce a driving-force contributing to the change of metabolic pathways and osmolyte production. In the present study, the enhancement of N-components as soluble proteins and amino acids may be ascribed to the magnetic energy experienced by dry seeds during the exposure duration that could trigger the enzymes systems (i.e., esterases) and activate the metabolism of plants [58]. This was confirmed, herein, by recording notable activation of N-metabolizing enzyme, NR activity, under different magnetic field intensities in comparison to the seeds without SMF. These novel proteins produced in magnetized plants may offer a storage type of nitrogen, which is used under unfavorable conditions and osmotic regulation. Furthermore, the production of proline (a protective osmolyte) is vastly exacerbated by SMF, thus reinforcing cellular structures via decreasing osmotic potential and maintaining protein against denaturation and ROS scavenging [59,60]. Thus, the increase of activities of certain enzymes is another action mechanism that could be involved in the biological response to magnetic treatments.

The activation of the NR pool by seeds magnetization also affects NO production. As NR is a fundamental pathway of NO production, the regulation effect of magnetization on plant development was noted by the accumulation of NO production, especially SMF2 and SMF3. Earlier, Kataria et al., [61] stated the striking function of NR in the production of NO in response to the magneto-priming of soybean to salt tolerance. This enhancer effect of SMF via NO could be associated with regulation various biochemical pathways as interaction with phytohormones (as modulation auxin content in roots), capturing of ROS (as peroxide and superoxide), attenuation of lipid peroxidation, membrane damage, and the activation of antioxidative responses [62]. In addition to NO, H_2_S is another endogenous gasotransmitter and a signaling molecule that also followed in lettuce leaves exposed to SMF. The regulatory mechanisms coined for pre-sowing exposure to magnetic therapy involving H_2_S accumulation, which is involved in plant growth and development, seed germination, root organogenesis, stomatal closure, and plant maturation [63]. Also, H_2_S is one of the pools related to the stimulation of the production of NO and the interplay between H_2_S and NO modulate plant growth and development [64]. So, it is reasonable to conclude that SMF may enhance NO and H_2_S, which triggers a cascade of biochemical procedures that affect the growth of lettuce plants.

Oxidative damage is a result of oxidation of macromolecules such as lipids, proteins, DNA, etc. via ROS are exceptionally reactive byproducts that cause the damage of membranes and the prevention of plant growth [65,66,67,68]. In this study, the reduction of H_2_O_2_ and O_2_^−^ content by SMF confirmed that the magnetized plants were capable of controlling the production of ROS. As lipid peroxidation is usually applied as a clear notion of oxidative stress, the decrement of MDA, herein, refers to non-oxidative burst ability of magnetized lettuce plants and, hence, the reliability of membranes. In this regard, MF is claimed to affect plasma membrane structure and function via polarization of dipoles and ionic activity [69]. In conformity, Poinapen et al. [70] observed that the magnetic field improves the order of lipids in plasma membranes causing intact membrane structure through the reduction in the fluid-structure of membrane lipids. The data of the present investigation revealed that apoplastic components may act as redox regulators signaling under SMF. In this sense, the magnetic field has a potential effect on the antioxidant system of the cells, and MF is implicated in antioxidant-modulated response in the apoplast, causing tight control of the disturbance in the redox status [71]. Overall, the constructive impact of SMF on lettuce plant growth may be due to keeping equilibrium between production and scavenging of ROS.

Scavenging of excess ROS is achieved by an effective arsenal of antioxidant mechanisms. This antioxidant system comprises enzymatic and non-enzymatic components to suppress ROS, and it functions at various subcellular partitions include peroxisomes, chloroplasts, plasma membranes, and endoplasmic reticulum. Enzymatic antioxidants include enzymes SOD, CAT, GPX, APX, GPX, GST, glutathione reductase, monodehydroascorbate reductase, and dehydroascorbate reductase and non-enzymatic antioxidants involve phenolic, carotenoids, ascorbate, glutathione, proline, glycine betaine, flavonoids, and tocopherols [66]. In this context, SOD breaks down superoxide radicals into oxygen and H_2_O_2_ [72] and was found to be enhanced by magneto-priming which kept the superoxide anion at a level lower than control plant. The antioxidative machinery motivated by magnetism is also witnessed via a vast array of hydrogen peroxide catabolizing enzymes such as CAT and the peroxidases family, which kept H_2_O_2_ lower than in the control. Of that machinery, CAT disintegrates H_2_O_2_ into water and oxygen, thus inhibiting cellular damage in plants [73]. Peroxidase family as APX is considered one of the most extensively dispersed antioxidant enzymes in plant cells and APX isoforms have an upper attraction for H_2_O_2_ than CAT using ascorbate as substrate [74], POD is another peroxidase utilizes guaiacol and pyrogallol as substrates for H_2_O_2_ detoxification, and GPX acts as an operative scavenger of reactive intermediate types of peroxyl radicals and superoxide radical as well as modulation of cellular redox balance by modulating the thiol-disulfide homeostasis [72]. The data unequivocally declared that the significant increase in GSTs activity under magnetism associated with the low oxidative stress capacity in plant tissues. These antioxidative enzymes were significantly exacerbated in the leaves derived from magnetic-primed seeds.

The ROS-scavengers aiding complementary agents comprised of small molecular weight non-enzymatic antioxidants were also triggered by SMF applied. Ascorbate, reduced glutathione, and α-tocopherol promotion is a crucial notion of magnetic field pre-sowing exposure. In this regard, ascorbate is a potent antioxidant due to its ability to contribute electrons in some of the non-enzymatic and enzymatic reactions. Ascorbate has been revealed to play a chief role in some physiological procedures in plants, including differentiation, metabolism, growth, and a potent free radical scavenger [66,68]. Besides, tocopherols are a cluster of lipophilic antioxidants that contributes to the detoxification of lipid peroxyl radicals, oxygen-free radicals, and ^1^O_2_ [66]. Reduced glutathione acts as a donor of reduced S during secondary metabolites production, and glutathione is involved in signaling processes, biosynthetic streams, ROS-detoxification, antioxidant biochemistry, and redox homeostasis [75] besides being a substrate of GPX and GST enzymes. Thus, magnetism promotes the defense line against ROS production and kept the cells less energized, thus keeping optimum metabolic pools for suitable cell functioning. Similar inductions in the activity of the antioxidant enzymes in plants under SMF treatments have also been reported in wheat [76] and soybean [77].

In the present investigation, the data for PPO activity were dissimilar from the other antioxidant enzymes. PPO, as an oxidizer of phenolic compounds [78], is not induced by magnetic treatment but rather declined in its content compared to non-treated plants. Besides, Wang et al. [74] stated that the stimulation of oxidizing enzymes like PPO in leafy vegetables as lettuce reduces the crop quality, so the reduction of PPO as reported herein, may enhance the nutrition value of magneto-primed lettuce seedlings. Therefore, alteration in non-enzymatic and enzymatic antioxidants by SMF was complex and might be correlated to the intensity of SMF.

The magnetic field is bestowed with a myriad of antioxidants that increase the quality of lettuce leaves and hence their nutritional value. A further sign of the induction of health value of the magnetized lettuce leaves, via activation of the secondary metabolism pool compared to non-magnetized plants. This further mechanism was emphasized vastly via activation of PAL activity, which is the main pathway of phenol-related compounds production. SMF enhanced the content of phenolic compounds and anthocyanin in almond seeds [79], and these results are agreement with our findings. The activation of PAL in response to magnetic therapy was mirrored by the accumulation of phenolic compounds that function as the strong non-enzymatic antioxidant and quench free radicals in cells [80]. Ahmad et al. [81] proposed that magnetic field influence on cryptochrome-dependent responses in *Arabidopsis*. In plants, cytochromes effect on various features of growth and development, i.e., participation in de-etiolation reactions such as hypocotyl growth retardation and leaf and cotyledon expansion [82] and anthocyanin accumulation. Thus, the accumulation of anthocyanin, herein, is a response of lettuce plants to MF and a major supportive role in the way of lettuce regulations of growth and nutrition status. The similar positive response of flavonoids as a bioactive agent and a part of phenolic compounds to magnetism was also documented in the current work, which supports the antioxidant properties to magnetic-field-submitted plants. These compounds stabilize membranes by decreasing their fluidity, which in turn limits the diffusion of free radicals and reduces the peroxidation of membrane lipids. Stabilization of the membrane is due to phenolics ability (especially flavonoids) to bind to some of the integral membrane proteins and phospholipids [80,83]. This may have partially accounted for the maintaining steady state of lipid peroxidation in plants treated by the magnetic therapy, hence higher membrane integrity compared to control plants. In the present research, SOD, CAT, APX, GPX, POD, and GST activities and non-enzymatic antioxidative including phenol, flavonoid, anthocyanin, tocopherol, ascorbate, and glutathione were witnessed to augment under SMF treatment. The increments of this antioxidative system by SMF work synergistically to regulate ROS and might be considered as a crucial clue for the potential enhancement in the growth of lettuce in plants derived from magnetically exposed seeds. These regulatory mechanisms could have effects on late plant development as an indirect effect of the initial magnetic stimulation. Thus, the highest enhancement of lettuce crop yield, which reached more than 50% over the control value at SMF3, and the lowest increase in the yield by 17% over the control are the net result of all the metabolic regulations under different intensities.

## 5. Conclusions

SMF stimulated the growth and biomass production of lettuce plants. This positive impact of SMF on lettuce was associated with the enhancement of osmoregulation substances, secondary metabolites, stimulation of the ROS scavenging system via the improvement of enzymatic and non-enzymatic antioxidants and hence the decrement of lipid peroxidation, thereby improving the quality of lettuce leaves. These regulations can be translated as the nutraceutical quality of a crop by its antioxidant content. Thus, the consumption of lettuce as a food with high nutrition value of primary and secondary metabolites and antioxidants that participate in the preservation of cells against oxidative burst frustrates many degenerative diseases. Further, the beneficial effect of SMF exposure is not limited to morphogenesis and biochemical responses but extended to the yield. The data also demonstrates that no malformation or abnormal changes were detected in the yield produced by SMF, so magnetic therapy is a commercially prosperous agricultural practice, and magneto-priming can be used as an adequate environmentally friendly physical method.

## Figures and Tables

**Table 1:** Effect of different static magnetic field (SMF) intensities for three exposure periods on the shoot and root length, shoot and root dry weight (DW), crop yield, number of lateral roots, and stem thickness of lettuce (*Lactuca sativa)* seedlings. SMF0 (control), SMF1 (0.44 T), SMF2 (0.77 T), SMF3 (1 T), and h (hour).

Treatments	Shoot Length (cm Plant^−1^)	Root Length (cm Plant^−1^)	Shoot DW (mg Plant^−1^)	Root DW (mg Plant^−1^)	Crop Yield (ton/hectare)	Number of Lateral Roots	Stem Thickness (mm)
**MF0**	7.27 ± 0.24 ^c^	5.16 ± 0.16 ^e^	10.33 ± 0.33 ^e^	3.01 ± 0.12 ^c^	57.69 ± 1.61 ^g^	17 ± 0.57 ^d^	1 ± 0.05 ^ab^
**MF1+ 1 h**	8.50 ± 0.23 ^b^	8.00 ± 0.10 ^b^	21.01 ± 0.57 ^c^	8.14 ± 0.1 ^b^	67.98 ± 1.24 ^f^	22 ± 0.57 ^c^	1 ± 0.06 ^ab^
**MF1+ 2 h**	8.50 ± 0.28 ^b^	7.00 ± 0.15 ^c^	31.11 ± 0.57 ^ab^	12.33 ± 0.37 ^a^	75.73 ± 1.71 ^e^	28 ± 0.68 ^b^	1.5 ± 0.08 ^ab^
**MF1+ 3 h**	8.00 ± 0.12 ^b^	9.02 ± 0.08 ^a^	16.01 ± 0.51 ^d^	7.19 ± 0.26 ^b^	76.11 ± 1.52 ^de^	30.7 ± 0.33 ^ab^	1.5 ± 0.07 ^ab^
**MF2+ 1 h**	8.00 ± 0.10 ^b^	8.00 ± 0.10 ^b^	22.03 ± 0.55 ^c^	8.37 ± 0.33 ^b^	75.14 ± 1.41 ^e^	28 ± 0.57 ^b^	2 ± 0.09 ^a^
**MF2+ 2 h**	8.50 ± 0.15 ^b^	8.00 ± 0.11 ^b^	23.66 ± 0.33 ^c^	8.67 ± 0.29 ^b^	81.37 ± 1.37 ^c^	28 ± 0.56 ^b^	2.5 ± 0.10 ^a^
**MF2+ 3 h**	8.00 ± 0.17 ^b^	8.00 ± 0.13 ^b^	28.12 ± 0.59 ^b^	12.23 ± 0.36 ^a^	84.99 ± 1.32 ^b^	32.6 ± 0.54 ^a^	2.5 ± 0.12 ^a^
**MF3+ 1 h**	9.20 ± 0.15 ^a^	7.00 ± 0.05 ^c^	35.66 ± 0.88 ^a^	12.67 ± 0.35 ^a^	78.88 ± 0.94 ^d^	33 ± 0.75 ^a^	2 ± 0.08 ^a^
**MF3+ 2 h**	8.00 ± 0.28 ^b^	7.00 ± 0.28 ^c^	17.00 ± 0.57 ^d^	6.66 ± 0.32 ^bc^	84.88 ± 1.19 ^b^	22.7 ± 0.83 ^c^	2 ± 0.06 ^a^
**MF3+ 3 h**	8.00 ± 0.10 ^b^	6.00 ± 0.10 ^d^	17.01 ± 0.58 ^d^	5.03 ± 0.24 ^bc^	90.49 ± 0.82 ^a^	23.6 ± 0.32 ^c^	1 ± 0.04 ^ab^

Different letters indicate statistically significant values following Duncan’s multiple range test at *p* < 0.05. Each value represents the mean of three (*n* = 3) replicates ± standard errors (SEs).

**Table 2:** The effect of different intensities of the static magnetic field (SMF) for three exposure periods on proline, total soluble sugars, total soluble proteins, and total free amino acids of lettuce (*Lactuca sativa*) seedlings. SMF0 (control), SMF1 (0.44 T), SMF2 (0.77 T), SMF3 (1 T), and h (hour).

Treatments	Proline (µg g^−1^ FW)	Total Soluble Sugars (mg g^−1^ FW)	Total Soluble Proteins (mg g^−1^ FW)	Total Free Amino Acids (mg g^−1^ FW)
**MF0**	0.84 ± 0.03 ^d^	56.76 ± 0.69 ^e^	31.88 ± 1.23 ^d^	4.81 ± 0.46 ^e^
**MF1+ 1 h**	1.60 ± 0.02 ^c^	107.39 ± 1.31 ^b^	39.47 ± 0.36 ^d^	10.61 ± 0.68 ^a b^
**MF1+ 2 h**	1.49 ± 0.06 ^c^	100.37 ± 1.22 ^b c^	36.30 ± 0.71 ^d^	9.92 ± 0.72 ^c^
**MF1+ 3 h**	2.69 ± 0.03 ^b^	118.72 ± 1.44 ^a^	61.45 ± 0.57 ^b c^	11.74 ± 0.69 ^a^
**MF2+ 1 h**	4.19 ± 0.09 ^a^	85.88 ± 1.04 ^c^	52.70 ± 0.49 ^c^	8.49 ± 0.41 ^c d^
**MF2+ 2 h**	4.95 ± 0.08 ^a^	110. 17 ± 1.42 ^b^	98.39 ± 0.92 ^a^	10.89 ± 0.91 ^a b^
**MF2+ 3 h**	2.06 ± 0.04 ^b^	116.37 ± 0.84 ^a^	36.21 ± 0.31 ^d^	11.51 ± 0.84 ^a^
**MF3+ 1 h**	3.12 ± 0.09 ^ab^	69.89 ± 1.10 ^d^	45.28 ± 0.42 ^c^	6.91 ± 0.81 ^d e^
**MF3+ 2 h**	1.35 ± 0.07 ^c^	90.88 ± 0.69 ^c^	73.67 ± 0.68 ^b^	8.98 ± 0.72 ^c d^
**MF3+ 3 h**	0.84 ± 0.02 ^d^	56.32 ± 1.14 ^e^	35.55 ± 0.33 ^d^	5.61 ± 0.56 ^d e^

Different letters indicate statistically significant values following Duncan’s multiple range test at *p* < 0.05. Each value represents the mean of three (*n* = 3) replicates ± standard errors (SEs).

**Table 3:** Effect of different intensities of the static magnetic field (SMF) for three exposure periods on proline, total flavonoid, total phenol, ascorbic acid (ASA), reduced glutathione (GSH), a-tocopherol contents and Phenylalanine ammonia-lyase (PAL) activity in lettuce (*Lactuca sativa*). SMF0 (control), SMF1 (0.44 T), SMF2 (0.77 T), SMF3 (1 T), and h (hour).

Treatments	Anthocyanins (µg g^−1^ FW)	Flavonoids (mg g^−1^ FW)	Phenolics (mg g^−^^1^ FW)	ASA (μg g^−^^1^ FW)	GSH (µmol g^−1^ FW)	*α*-Tocopherol (μg g^−^^1^ FW)	PAL (μmol mg^−^^1^ Protein^−^^1^ min^−^^1^)
**MF0**	0.07 ± 0.002 ^h^	1.27 ± 0.073 ^g^	3.08 ± 0.18 ^f^	22.87 ± 0.76 ^h^	6.71 ± 0.23 ^e^	304.63 ± 12.73 ^f^	44.36 ± 1.42 ^g^
**MF1+ 1 h**	0.09 ± 0.001 ^g^	2.51 ± 0.40 ^e^	9.62 ± 0.17 ^b^	32.65 ± 0.38 ^e^	9.58 ± 0.11 ^d^	739.42 ± 16.57 ^b^	66.29 ± 6.14 ^d e^
**MF1+ 2 h**	0.10 ± 0.002 ^g^	3.47 ± 0.033 ^c^	8.87 ± 0.16 ^c^	41.67 ± 0.49 ^d^	12.23 ± 0.14 ^c^	400.14 ± 13.57 ^e^	70.68 ± 1.76 ^d^
**MF1+ 3 h**	0.12 ± 0.001 ^e^	3.49 ± 0.032 ^c^	10.95 ± 0.19 ^a^	45.86 ± 0.54 ^c^	13.45 ± 0.18 ^c^	777.05 ± 21.16 ^a b^	64.38 ± 1.75 ^d e^
**MF2+ 1 h**	0.14 ± 0.003 ^d^	2.86 ± 0.026 ^d^	6.82 ± 0.12 ^e^	40.13 ± 0.47 ^d^	11.77 ± 0.13 ^c^	493.89 ± 14.65 ^d^	94.98 ± 2.34 ^b^
**MF2+ 2 h**	0.12 ± 0.004 ^e^	3.95 ± 0.037 ^b^	6.41 ± 0.11 ^e^	72.17 ± 0.85 ^a^	22.18 ± 0.25 ^a^	364.71 ± 15.65 ^e^	101.64 ± 2.45 ^a^
**MF2+ 3 h**	0.25 ± 0.003 ^a^	2.92 ± 0.027 ^d^	8.72 ± 0.51 ^c^	26.61 ± 0.31 ^g^	7.81 ± 0.09 ^d e^	807.26 ± 12.43 ^a^	87.84 ± 1.45 ^c^
**MF3+ 1 h**	0.18 ± 0.004 ^c^	2.00 ± 0.018 ^f^	6.86 ± 0.12 ^e^	24.74 ± 0.29 ^h^	7.26 ± 0.12 ^d e^	772.05 ± 16.97 ^a b^	55.04 ± 2.30 ^f^
**MF3+ 2 h**	0.22 ± 0.003 ^b^	4.86 ± 0.045 ^a^	6.71 ± 0.14 ^e^	53.91 ± 0.64 ^b^	15.79 ± 0.16 ^b^	784.60 ± 17.97 ^a^	61.53 ± 1.92 ^e^
**MF3+ 3 h**	0.11 ± 0.005 ^f^	3.53 ± 0.033 ^c^	7.39 ± 0.13 ^d^	29.53 ± 0.35 ^f^	8.66 ± 0.10 ^d^	673.03 ± 15.98 ^c^	50.34 ± 1.50 ^f g^

Different letters indicate statistically significant following Duncan’s multiple range test at *p* < 0.05. Each value represents the mean of three (*n* = 3) replicate ± standard errors (SEs).
